# One-Year Multicenter Randomised Controlled Trial Comparing OT Equator® and Locator Attachments to Retain an Early Loaded Implant Overdenture on Two Implants

**DOI:** 10.1155/2023/2745262

**Published:** 2023-07-07

**Authors:** Marco Tallarico, Luca Fiorillo, Marco Montanari, Roberto Scrascia, Corina Marilena Cristache, Emiliano Ferrari, Alessio Casucci, Erta Xhanari, Saturnino Marco Lupi, Irene Ieria, Edoardo Baldoni, Ruggero Rodriguez y Baena, Gabriele Cervino

**Affiliations:** ^1^School of Dentistry, Sassari 07100, Italy; ^2^School of Dentistry, Department of Biomedical and Dental Sciences and Morphofunctional Imaging, University of Messina, Via Consolare Valeria 1, Messina 98125, Italy; ^3^School of Dentistry, Aldent University, Tirana 1001, Albania; ^4^Multidisciplinary Department of Medical-Surgical and Odontostomatological Specialties, University of Campania “Luigi Vanvitelli”, Naples 80121, Italy; ^5^Department of Prosthodontics, Dr D. Y. Patil Dental College and Hospital, Dr D. Y. Patil Vidyapeeth, Pimpri, Pune 411018, India; ^6^Private Practice, Cesena 41121, Italy; ^7^Independent Researcher, Taranto 74121, Italy; ^8^Faculty of Midwifery and Medical Assisting, “Carol Davila” University of Medicine and Pharmacy, Bucharest 020021, Romania; ^9^Independent Researcher, Bologna 40123, Italy; ^10^Independent Researcher, Montepulciano Stazione 53045, Italy; ^11^Dental Clinic, University of Pavia, Pavia 27100, Italy; ^12^Independent Researcher, Rome 00151, Italy

## Abstract

This investigation aimed to compare the effectiveness of the OT Equator® (Rhein, Bologna, Italy) and the Locator attachment systems used to retain early loaded implant-retained overdentures. This study was designed as a multicenter randomised controlled trial of parallel groups. After implant placement, the patients were randomised to receive OT Equator® attachments in the test group or Locator attachments in the control group. The outcome measures were implant and prosthetic success and survival rates, any biological and technical complication, marginal bone loss, patients' satisfaction, and periodontal parameters. Overall, 42 patients were consecutively enrolled and treated. One implant was lost in the control group, while no implants were lost in the test group. No prostheses failed in both groups. Only a few complications were experienced in both groups. The main was represented by loss of retention of the attachments (retentive caps). The OT Equator® attachment showed statistically lower periodontal parameters. In conclusion, both attachment systems were suitable for overdenture implant retention.

## 1. Introduction

Ageing is accelerating globally, resulting in an increased number of dental implants placed in patients who are elderly and who may require healthcare support due to systemic problems. Implant rehabilitation in geriatric patients is a viable treatment option with a high implant survival rate. However, a recent systematic review from the University of Geneva, Switzerland, clearly concluded that age alone should not be a limiting factor for dental implant therapy [1]. Substantial evidence supports predictable, long-term successful implant therapy in elderly patients in terms of implant survival rates, patient satisfaction, periodontal parameters, and complications. There are several evidence-based rehabilitation procedures for elderly patients, including, but not restricted to, dental implant therapy. As per the McGill consensus statement, implant-supported overdentures have been established as a conventional approach for prosthetic management of fully edentulous jaws, whether employing immediate or delayed loading procedures [2]. Clinical evidence has demonstrated that utilising two-to-four implants to support mandibular overdentures can effectively address the needs of individuals with missing teeth, ensuring good retention and support [3, 4]. The crucial factor in achieving favourable outcomes lies in the secure attachment of dental implant overdentures. The McGill consensus statement states that technological advancements have significantly enhanced implants' affordability, effectiveness, ease of placement, and long-term success. Nevertheless, the rising expectations for improved aesthetics and functionality among patients and the challenges posed by atrophic conditions have made developing comprehensive removable solutions more challenging than in the past.

To face economically viable rehabilitation, implant overdentures have evolved, not only improving the aesthetics of the teeth but also supporting materials. Ageing significantly increased the microhardness values of resins, resulting in chromatic alteration [5]. For several years, retentive anchors with a titanium matrix and ball-type attachment systems were considered the better choice from a financial point of view, considering the initial low cost of the components and the reduced number of complications [6]. Nevertheless, attachment systems evolved from a ball type to a low-profile type, aimed at improving the retention and stability of overdentures. Among the latter, the OT Equator® (Rhein'83, Bologna, Italy) attachment system is becoming a suitable attachment, ensuring good retention of the implant overdenture. The main benefit of the OT Equator® (Rhein'83 Bologna, Italy) attachment system is its reduced shape and volume, compared to the well-known Locator attachment (Zest Anchors LLC, Espandido, CA, USA) [7, 8]. Moreover, the option for fixed and removable dentures makes this attachment unique [9, 10].

This multicenter randomised controlled trial aimed to compare peri-implant tissue health, complications, and patient preference between implant-retained overdentures delivered on two unpainted implants with different attachment systems, Locator and OT Equator® (Rhein'83 Bologna, Italy). The null hypothesis is that there are no differences in clinical outcomes.

## 2. Materials and Methods

This study was conducted following the principles outlined in the Helsinki Declaration of 1964 for biomedical research involving human subjects, as amended in 2013, and received ethical approval by the Ethical Committees of the Aldent University of Tirana (4/2018). This study was designed as a multicenter randomised controlled trial of the parallel group with two arms. Patients were enrolled and consecutively treated in eight European centers between December 2017 and November 2018. Each patient was provided with appropriate information regarding the study's nature. Written consent forms encompassing surgical and prosthetic procedures and clinical and radiological data utilisation were obtained from all individuals involved. The present research has been registered in ClinicalTrials.gov (NCT03640910), and the manuscript was written according to the CONSORT guidelines.

Any healthy individual (ASA 1 and 2 classifications; American Society of Anesthesiologists, https://www.asahq.org), aged 18 years or older at the time of enrollment, with complete edentulism in the mandible, or a failing dentition in the mandible, in need of an implant-retained overdenture, was considered eligible for the study. The exclusion criteria are reported in Table 1.

Preoperative photographs, panoramic X-rays, and periodontal status were obtained for initial screening and evaluation. Hopeless teeth (in the mandible) were extracted three months before implant placement. All patients received a temporary complete removable denture before implant placement, according to functional and esthetic requirements. Nevertheless, if patients and clinicians accurately judged the complete removable denture, it was used as a temporary solution.

On the day of the surgery, a single dose of an antibiotic (two g of amoxicillin or 500 mg of azithromycin if allergic to penicillin) was administered one h before implant placement. Immediately before surgery, the participants rinsed with a 0.2% chlorhexidine mouthwash for one minute. Local anaesthesia preferred by the surgeon was administered. The minimally invasive mucoperiosteal flap was elevated. Implants were placed in the mandible's interforaminal region (canine region) using a previously reported surgical approach [11]. Any brand of implants that provide either OT Equator® (Rhein'83, Bologna, Italy) or Locator (Zest Anchors LLC, Espandido, CA, USA) attachments was used. The preoperative radiographs and study models dictated the implant lengths and diameters. Implants were placed free-hand with the aid of parallel implant pins.

After surgery, the patients were instructed to avoid any trauma at the surgical site, including brushing procedures. A postsurgical cold and soft diet was prescribed. Postoperatively, smokers were advised to abstain from smoking for two weeks. Detailed oral hygiene instructions were provided, including the recommendation of rinsing three times a day with 0.12% chlorhexidine. Analgesics, such as 600 mg of ibuprofen or similar alternatives, were prescribed as necessary. Sutures were typically removed within a timeframe of ten to fourteen days. According to a previously reported workflow, the prosthetic procedures began eight weeks after implant placement [11]. According to an early loading protocol, a new metal-reinforced, complete removable denture was delivered in both groups within four weeks after implant placement. After two to three days, the healing abutments were unscrewed, and the attachments were connected chairside to the new removable prosthesis. The patients were randomised to receive OT Equator®® attachments (Rhein'83, Bologna, Italy) in the test group or Locator attachments (Zest Anchors LLC) in the control group. Randomised attachment systems were placed and tightened according to the manufacturer's instructions.

**Figure 1:**
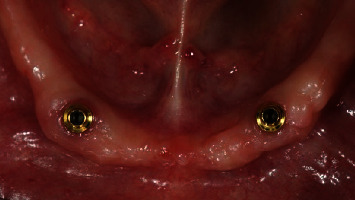
Test group: (OT Equator®) one month after implant placement, occlusal view.

**Figure 2:**
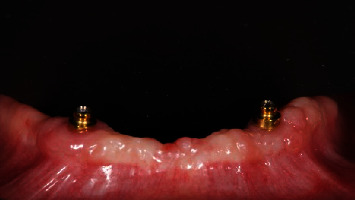
Test group: (OT Equator®) one month after implant placement, frontal view.

**Figure 3:**
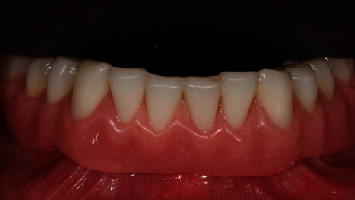
Test group: definitive prosthesis one year after implant placement, frontal view.

**Figure 4:**
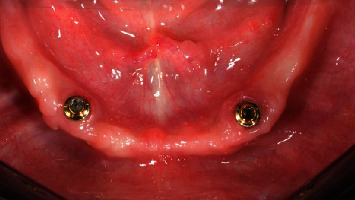
Test group: (OT Equator®) one year after implant placement, occlusal view.

**Figure 5:**
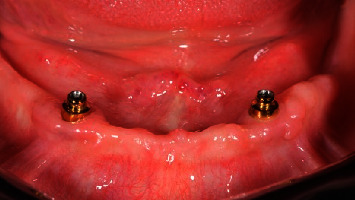
Test group: (OT Equator®) one year after implant placement, frontal view.

In the test group (Figures 1–5), after gingival healing, the newest low-profile OT Equator®® attachments were screwed onto the implants using the OT Equator® square screwdriver, with a 22–25 N cm torque range. The cuff heights ranged from 0.5 to 7.0 mm, depending on the size of the transition zone of each implant, easily measured using the colour-coded millimetre cuff height measurer gauge (Rhein'83, Bologna, Italy) after healing abutment removal. Afterwards, the needed space to accept the female housing steel cage was prepared in the fitting surface of the removable complete mandibular denture. Silicone protective discs (Rhein'83, Bologna, Italy) were placed over the OT Equator® attachments. Extra-soft (yellow, 600 g) retentive caps were initially placed into the female steel housing, attached to OT Equator®, and finally fixed to the denture using self-cured acrylic resin. At the same time, the patient held the dentures in occlusion, directly chairside. After complete polymerisation, the denture was picked up, and silicone discs were removed. Acrylic excess was trimmed, and the denture was refined and polished. One month after the delivery of prostheses, the yellow retentive caps were replaced with a more robust type (pink, 1200 g).

**Figure 6:**
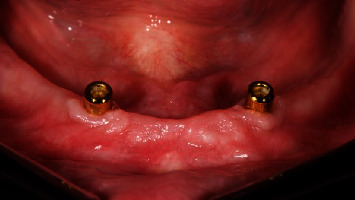
Control group: (Locator) one month after implant placement, occlusal view.

**Figure 7:**
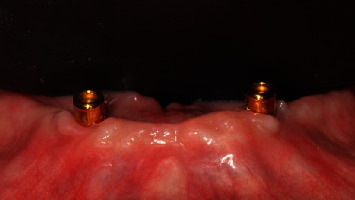
Control group: (Locator) one month after implant placement, frontal view.

**Figure 8:**
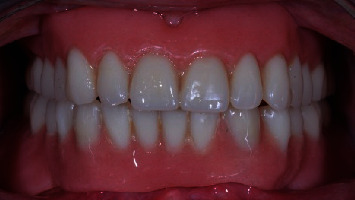
Control group: (Locator) prosthesis one year after implant placement, frontal view.

**Figure 9:**
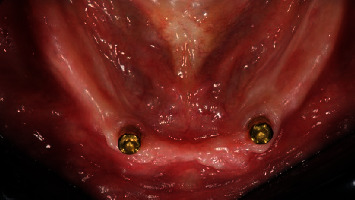
Control group: (Locator) one year after implant placement, occlusal view.

**Figure 10:**
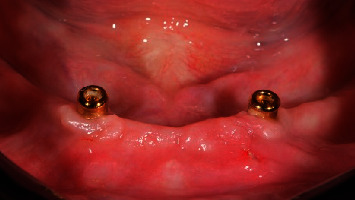
Control group: (Locator) one year after implant placement, frontal view.

In the control group (Figures 6–10), the Locator attachment (Zest Anchors LLC) was screwed onto the implants using the Locator screwdriver (Zest Anchors LLC), with a torque range of 20–25 Ncm. The cuff heights of 2.5 or 4.0 mm, depending on the size of the transition zone of each implant, were measured using the deep probe of the implant line after healing abutment removal. Afterwards, spaces to accept the female housing steel cage were prepared in the fitting surface of the removable complete mandibular denture. Silicone protective white rings (Zest Anchors LLC) were placed over the Locator attachments. Passive black caps were used to load the attachment, attached to Locator, and finally fixed to the denture using self-cured acrylic resin, while the patient held the dentures in occlusion, directly chairside. After complete polymerisation, the denture was picked up, and white rings were removed. Acrylic excess was trimmed, and the denture was refined and polished. Black caps were removed, and blue ones (6N) were mounted in the steel housing. One month after the delivery of prostheses, the retentive caps were replaced with a pink matrix (12N).

Occlusion was developed in both groups to deliver lingualised occlusion with balanced contacts during function, avoiding premature contact. Nevertheless, when the opposing arch was a completely removable denture, the over-jet had to be left purposely broad, from 2 to 5 mm, to avoid interferences during function. Domiciliary oral hygiene instructions were given to both groups. These depend on residual dentition. However, in all cases, instructions were given to clean attachments and prostheses. Follow-up visits were scheduled for occlusal adjustments and oral hygiene quality control every six months and every year for retentive cap replacement [11].

### 2.1. Outcomes

The outcome measurements were implant and prosthetic success and survival rates, biological and technical complications, marginal bone loss, patients' satisfaction (Oral Health Impact Profile, OHIP-22), and periodontal parameters (bleeding index (BI) and plaque index (PI)):

(i) An implant was deemed unsuccessful if it exhibited any form of mobility, determined by tapping or rocking the implant head using metallic instruments. In addition, progressive marginal bone loss, infection, or any mechanical issues that rendered the implant nonfunctional, despite maintaining stability within the bone, were also regarded as factors indicating implant failure.
(ii) The prosthodontic success of implant overdentures was assessed with the six-field table analysis proposed by Payne and coworkers (Table 2) [12–14].
(iii) Complications: Any biological (pain, swelling, suppuration, etc.) and mechanical (attachment loosening, fracture of the prosthesis, loss of the retention, etc.) complications were evaluated. In particular, loss of retention was assessed by the patient and the clinician with the loss of the snap-fit sound on complete seating, and in general, evaluating the force to be applied until the dentures detached. After that, a microscopic evaluation (10x magnification) was used to confirm the wear and/or tear of the retentive caps.
(iv) Marginal bone levels were evaluated utilising intraoral digital or conventional periapical radiographs, following a protocol outlined in prior studies. Two trained examiners, partially blinded to the study, independently assessed the marginal bone levels on each periapical radiograph. They measured from the mesial and distal margins of the implant neck to the most coronal point where bone contact with the implant was observed. Marginal bone loss was determined by calculating the disparity between borderline bone levels at different intervals.
(v) The Oral Health Impact Profile (OHIP-19) questionnaire assessed the quality of life which the participants completed. A blinded examiner administered the questionnaire before treatment and one month and one year after the final prosthesis delivery. The questionnaire consisted of seven subscales FL = functional limitation, P1 = physical pain, P2 = psychological discomfort, D1 = physical disability, D2 = psychological disability, D3 = social disability, and *H*=handicap, with two to three questions each. Participants chose from five possible responses for each question: 4 = very often, 3 = fairly often, 2 = occasionally, 1 = hardly ever, and 0 = never/do not know. Lower OHIP total scores suggest improved oral health-related quality of life [12].
    FL 1. Have you had difficulty chewing any foods because of problems with your teeth, mouth, or dentures?
    FL 2. Have you had food catching in your teeth or dentures?
    P1 3. Have you had painful aching in your mouth?
    P1 4. Have you found it uncomfortable to eat any foods because of problems with your teeth, mouth, or dentures?
    P1 5. Have you had sore spots in your mouth?
    FL 6. Have felt that your dentures have not been fitting properly?
    P1 7. Have you had uncomfortable dentures?
    P2 8. Have you been worried by dental problems?
    P2 9. Have you been self-conscious because of your teeth, mouth, or dentures?
    D1 10. Have you had to avoid eating some foods because of problems with your teeth, mouth, or dentures?
    D1 11. Have you been unable to eat with your dentures because of problems with them?
    D1 12. Have you had to interrupt meals because of problems with your teeth, mouth, or dentures?
    D2 13. Have you been upset because of problems with your teeth, mouth, or dentures?
    D2 14. Have you been a bit embarrassed because of problems with your teeth, mouth, or dentures?
    D3 15. Have you avoided going out because of problems with your teeth, mouth, or dentures?
    D3 16. Have you been less tolerant of your partner or family because of problems with your teeth, mouth, or dentures?
    D3 17. Have you been irritable with other people because of problems with your teeth, mouth, or dentures?
    H 18. Have you been unable to enjoy other people company as much because of problems with your teeth, mouth, or dentures?
    H 19. Have you felt that life in general was less satisfying because of problems with your teeth, mouth, or dentures?
(vi) The bleeding index and the plaque index were evaluated at four sites around each implant-abutment interface at the baseline and one year after loading examination with a dedicated periodontal probe.

### 2.2. Statistical Analysis

A priori sample size calculation was performed online (https://clincalc.com/stats/samplesize.aspx) based on a previous preliminary report [15], given: alpha 0.05, beta 0.2, and power 0.80. Twenty centers were involved with six patients each to improve the sample size by at least one-third. Of these, three patients were to be treated with two implants and OT Equator® (Rhein'83), and the same number of patients were to be treated with two implants and two Locators (Zest Anchors LLC). The total sample size was to be 44 patients for each group. Data were planned to be collected 1, 3, and 5 years after loading.

Eight computer-generated restricted randomisation lists were created. The randomisation codes were enclosed in sequentially numbered, identical, opaque, sealed envelopes. Envelopes were opened successively after implant placement. One investigator, not involved in the study, was aware of the randomisation sequence and could have access to the randomisation lists, which were stored on his password-protected laptop.

Statistical analysis was developed to find differences between groups. Data were recorded in a spreadsheet (Numbers for Mac OS X). A statistician with expertise in dentistry analysed the data using the same software. Descriptive analysis was conducted using mean ± standard deviation with a 95% confidence interval (CI) for numerical parameters. Fisher's exact test was employed to compare the proportions of dichotomous outcomes, such as implant failures, prosthesis failures, and complications. Unpaired sample *t*-tests were utilised to compare the means at the patient level for continuous results, including OHIP, marginal bone loss, BOP, and PI. All statistical analyses were conducted at the patient level, and a significance level of 0.05 was applied.

## 3. Results

Each center was supposed to enrol six patients, but after the study began, it was noted that only eight out of twenty centers could enrol patients. Overall, sixty-three patients were screened for eligibility, but only 42 participants were consecutively enrolled in the trial by the eight participating centers. In particular, Dr. Tallarico (Rome), Dr. Cristache (Bucarest), and Dr. Casucci (Siena) recruited six patients, Dr. Montanari Marco (Forlì), Dr. Scrascia (Taranto), Dr. Ferrari (Bologna), and Prof. Rodriguez (Pavia) recruited five participants, and Dr. Xhanari (Tirana) recruited four participants. However, no patients dropped out after randomisation. Reasons for not including the 21 excluded patients are reported in Table 3.

The primary baseline patients' and implant characteristics of the 42 patients that were randomised are presented in Tables 4 and 5. There were no apparent significant baseline imbalances between the two groups, except for more female and younger patients in group one (test group).

*Implant Failures.* At the one-year follow-up, one implant failed in the control group at center seven, while no implants were lost in the test group. The difference was not statistically significant (*P*=0.4286). The patient lost the implant in position 33 six weeks after implant placement. The implant was replaced six months later with no other complications/failures. In the meantime, the patient wore the prosthesis attached to only one implant.

*Prosthesis Failures.* At the one-year follow-up, no prostheses failed in both groups (*P*=1.0).

*Complications.* At the one-year follow-up, three difficulties were experienced in each group. A comparison of implants and prosthesis failures and complications is reported in Table 6. The difference was not statistically significant (*P*=1.0). The prosthesis was broken ten months after its delivery in the test group at center one. It was repaired chairside in 30 minutes. At centers two and seven, patients each showed an early loss of retention of the caps replaced chairside in five minutes. In the control group, at centers two and seven, three patients (one at center two and two at center seven) showed an early loss of retention of the caps replaced chairside in five minutes.

A comparison of the mean marginal bone loss, OHIP, mean BI, and PI are reported in Table 7. There was a statistically significant difference only for periodontal parameters with a lower value for OT Equator® (Rhein'83).

## 4. Discussion

Both attachment systems provide successful results when comparing main and test groups, with no statistically significant differences except for better periodontal parameters experienced with OT Equator® (Rhein'83) attachments. So the null hypothesis of no differences was partially accepted. OT Equator is the slightest attachment on the market. The total vertical footprint (male + female and container) is only 2.1 mm. The maximum width is *ø* 4.4 mm. This system offers many solutions; depending on the spaces, it is possible to plan various solutions for overdentures. Available in two versions, castable and prefabricated abutments in titanium nitride (TiN), they are made individually for all types and diameters of existing implants on the market. The height of the OT Equator abutment is seven different sizes; the minimum height depends on the implant platform for a maximum height of 7 mm. The retentive caps have four sealing levels; the degree of retention changes depending on the colour. These retentive caps must always be used with the appropriate containers.

Lately, more and more completely edentulous patients with atrophic mandible or maxilla require fixed rehabilitations. Nowadays, overdenture retained by implants is one of the best solutions to achieve an optimal masticatory and phonetic function and satisfy esthetic requests. In the present randomised multicenter controlled trial, two different attachment systems for overdenture were evaluated. In particular, the implant and prosthesis success and their survival rate, the biological and technical complications, the marginal bone loss, and the quality of life were assessed by the Oral Health Impact Profile (OHIP-19) questionnaire. As shown by Khalid et al. [16], an improvement in the patient's outcomes after implant therapy was estimated, independent of the type of implant attachments. These data agree with the data from the present study, where the OHIP was significantly reduced in both groups.

Unfortunately, several centers failed to enrol patients. Nevertheless, 42 patients were finally enrolled and randomised. The enrolled patients were treated in eight different clinical centers and divided into two groups depending on the attachment system used. The two groups were balanced, as demonstrated in Table 5, except for more females and younger patients in the test group. In the present study, and previous report, it seems that sex did not affect the results [15]; however, it appears that female patients required functional and esthetic oral rehabilitation more than male patients. On the contrary, clinical evidence documented sex differences in oral health. Women's oral health declines more rapidly than men's.

The results of the present study partially agree with those of previous research, reporting successful outcomes in both groups [15, 17], with no significant differences, except for lower periodontal parameters in the test group. In fact, regarding 42 patients, no prostheses failure happened, and one implant failure was reported. The main complication, equally presented in both groups, was the loss of retention of the prosthesis due to wear of the retentive caps. Also, the literature is described as a common mechanical problem, regardless of the type of attachment used [1, 15].

Previously, Nieves Mínguez-Tomás et al. [18] evaluated, in vitro, the retention capacity of Locator (Zest Anchors LLC) and OT Equator® (Rhein'83 Bologna, Italy) attachments, concluding that both systems had similar retention values. The complication experienced in both groups was the wear of the retentive caps that required their replacement. As reported in the literature [19], most technical difficulties may occur in the first year. Despite this being a standard feature for overdenture, in our research, both groups experienced early needs for retentive caps replacement. The authors believe that the incorrect insertion of the overdenture may explain this problem. Re-explaining the attachment method and changing the matrix with a more retentive one were enough to accomplish the problem.

At one year follow-up, no statistically significant difference was found for MBL between the groups. The only meaningful difference was lower bleeding and plaque indices in the test group. Identical results were found in a preliminary multicenter retrospective analysis on implant overdentures conducted by Tallarico et al. [15] after five years of examination. The attachment height from the gingival margin was controlled by selecting the attachment height in both groups. The correct height of the attachments was needed to ensure their cleanliness, notably easier brushing, and the proper connection with the female portion. Even if successful results were found in both groups, the OT Equator® attachments have a low vertical profile of only 2.1 mm, making them the most miniature attachment system on the market. Due to the narrow diameter of the OT Equator® attachment, a possible explanation for better periodontal results in the test group could be the platform switching effect that could be obtained even in smaller diameter implants, contributing to marginal soft tissue maintenance [11–14, 16, 20]. Nevertheless, several confounding factors exist. For the latter, further studies are needed to confirm this hypothesis.

In the present study, all the implants were early loaded. Further investigation should be conducted to evaluate the performance of attachment systems in case of the immediate loading procedure [21], also assessing risks and benefits, including patients' satisfaction. Another study [22] presents a randomised controlled trial comparing the outcomes of two-implant- and three-implant-supported overdentures for edentulous mandibles. The study evaluated implant and prosthetic success rates, complications, marginal bone loss, patient satisfaction, and peri-implant tissue health. A total of 34 patients were enrolled in the trial, with 14 in the three-implant group and 20 in the two-implant group. Two implants failed in the three-implant group at the one-year follow-up, while no implants were lost in the two-implant group. Three complications were experienced in the two-implant group, and one occurred in the three-implant group. However, the groups had no statistically significant differences in any of the evaluated outcomes.

No prosthesis failure was registered, and only one early implant failure occurred in the control group. The implant was reinserted without further problems. The main limitation of the present study was the small sample size because most of the involved centers needed to recruit patients; moreover, the status of opposing dentition must be described in detail. Chewing efficiency may affect the wear of retentive elements up to the loss of retention of an implant-retained overdenture.

## 5. Conclusions

With the limitation of the present multicenter randomised controlled trial, successful results were found in both groups, even if statistically significant better periodontal parameters were found in the test group. This abutment (OT) type shows significant clinical and biomechanical advantages compared to similar abutments. Indeed, the smaller footprint from a spatial point of view is a good advantage for the clinician and the dental technician. Furthermore, more excellent retention guarantees reliability and predictability of oral rehabilitations. Further studies with larger sample sizes and longer follow-ups are needed to confirm the results of this preliminary report.

## Figures and Tables

**Table 1:** Exclusion criteria.

General contraindications to oral surgery
Pregnant or nursing
Intravenous bisphosphonate therapy
Alcohol or drug abuse
Heavy smoking (≥20 cigarettes/day)
Radiation therapy to the head or neck region within the last five years
Parafunctional activity
Untreated periodontitis
Psychiatric therapy or unrealistic expectations
Immunosuppressed or immunocompromised
Lack of opposite occluding dentition/prosthesis
Acute infection in the area intended for implant placement
Need for bone augmentation
Poor oral hygiene and motivation (full-mouth bleeding on probing (BoP) and full-mouth plaque index [PI] higher than 25%)
Patients participating in other studies, if the present protocol cannot be properly followed
Patients referred only for implant placement or unable to be followed for three years
Allergy or adverse reactions to the restorative materials

**Table 2:** Criteria for prosthodontic success [17].

Success	No evidence of retreatment except for accepted maintenance (includes patrix activation/repair/replacement, matrix activation/repair/replacement, and asymptomatic peri-implant/interabutment mucosal enlargement not requiring excision). There was a limit of two replacements of either patrix or matrix in the first year and five replacements in 5 years and one reline of the overdenture base in five years
Survival	Survival: patient could not be examined directly, but the patient or another clinician confirms no evidence of retreatment except that described for a successful outcome
Unknown	Unknown (lost to follow-up): the patient could not be traced; surviving or successful implant overdenture removed to allow—to provision of a new overdenture, e.g., conversion to another overdenture design with additional implants or a fixed implant prosthesis using the same or additional implants
Deceased	Deceased: the patient died during the study period regardless of whether successful or surviving criteria were experienced before death
Retreatment (repair)	Retreatment (repair): treatment of implant overdentures and/or mucosa where marginal integrity and associated patrices/matrices are maintained irrespective of modifications as long as it continues as an implant overdenture. More than two replacements of either patrix or matrix in the first year or more than five replacements in the first five years. Includes replacement of worn or fractured overdenture teeth/fractured overdentures, relining of overdenture more than once in five years, or excision of patrix-associated mucosal enlargement as a result of infringement on the shoulder/undersurface of the patrix
Retreatment (replace)	Retreatment (replace): part or all of implant overdenture is no longer serviceable because of either loss of implants or irreparable mechanical breakdown

**Table 3:** Patients not included with reasons [14].

Center 1 (*n* = 9)	1 patient refused implants; 3 patients requested fixed restorations; 5 patients required implant-supported hybrid overdentures
Center 2 (*n* = 4)	2 patients were treated just 5-6 months ago; 2 patients required fixed restorations
Center 3 (*n* = 4)	4 patients were not included. They required fixed restorations, and 4–6 implants were inserted
Center 4 (*n* = 0)	None
Center 5 (*n* = 2)	1 patient refused implants; 1 patient required fixed restoration
Center 6 (*n* = 0)	None
Center 7 (*n* = 2)	2 patients refused implants
Center 8 (*n* = 0)	None

**Table 4:** Main patient and implant characteristics.

	Groups 1 (test group) (*n* = 24)	Group 2 (control group) (*n* = 18)	*P* value
Sex (M/F)	3/21	8/10	0.0329
Mean age (years)	67.5	75.2	0.0152
Smoke	2	0	0.4983
Bone quality type I/II	11/13	12/6	0.2214
Mean implant length (I) (mm)	10.0	10.5	0.2620
Mean implant diameter (I) (mm)	3.8	3.8	0.7477
Mean implant length (II) (mm)	10.1	10.6	0.2675
Mean implant diameter (II) (mm)	3.8	3.8	0.9900

**Table 5:** Mean implant length and diameter per group and brand.

Imp leng	Imp diam	Imp leng	Imp diam	Implant's brand
*Control group*				
10	4	10	4	Osstem Implant TSIII
10	4.5	10	4.5	Osstem Implant TSIII
11.5	3.5	11.5	3.5	Osstem Implant TSIII
11.5	3	11.5	3	Megagen AnyRidge
10	3	10	3	Megagen AnyRidge
12	4.1	12	4.1	Straumann STL
12	4.1	12	4.1	Straumann STL
10	4.1	10	4.1	Straumann STL
10	4	10	4	Osstem implant TSIII
11.5	4	11.5	4	Osstem implant TSIII
10	3.8	10	3.8	Sweden & Martina
11.5	3.8	11.5	3.8	Sweden & Martina
9	3.8	11	3.8	Winsix
11	3.8	11	3.8	Winsix
11	3.3	11	3.3	Winsix
9	3.8	9	3.8	Winsix
9	3.8	9	3.8	Winsix
10	4.1	10	4.1	Straumann STL
10.5	3.8	10.6	3.8	Mean values

*Test group*				
11.5	4	11.5	4	Osstem Implant TSIII
11.5	4	11.5	4	Osstem Implant TSIII
8.5	3.5	8.5	3.5	Osstem Implant TSIII
10	3.5	10	3.5	Megagen AnyRidge
11.5	3.5	11.5	3.5	Megagen AnyRidge
11.5	3	11.5	3	Megagen AnyRidge
10	4.5	11	4	Megagen AnyRidge
10	4	10	4	Megagen AnyRidge
11.5	4	11.5	4	Megagen AnyRidge
10	3.5	10	4	Osstem Implant TSIII
11.5	3.5	11.5	3.5	Osstem Implant TSIII
10	4	10	4	Osstem Implant TSIII
11.5	3.5	11.5	3.5	Osstem Implant TSIII
10	3.8	10	3.8	Sweden & Martina
5	4.1	5	4.1	Sweden & Martina
11.5	3.3	11.5	3.3	Sweden & Martina
11	3.8	11	3.8	Winsix
11	3.8	11	3.8	Winsix
9	3.8	11	3.8	Winsix
10	4.1	10	4.1	Straumann STL
10	4.1	10	4.1	Straumann STL
7	5	7	4	Osstem Implant TSIII
10	4	10	4	Osstem Implant TSIII
7	4	7	4	Osstem Implant TSIII
10	3.8	10.1	3.8	Mean values

**Table 6:** Comparison between the groups (primary outcomes).

	Group 1 (test group) (*n* = 24)	Group 2 (control group) (*n* = 18)	*P* value
Implant failure	0	1	0.4286
Prosthesis failure	0	0	1.0
Complications	3	3	1.0

**Table 7:** Comparison of MBL, OHIP, BI, and PI between the groups.

Mean values	Groups	Baseline mean ± SD	1-year mean ± SD	Difference mean ± SD (95% CI)	*P* value
MBL (mm)	Gr. 1 (*n* = 24)	0.00 ± 0.02	0.10 ± 0.08	0.09 ± 0.08	
	Gr. 2 (*n* = 18)	0.05 ± 0.08	0.13 ± 0.10	0.09 ± 0.16	
Difference				0.00 ± 0.038 (−0.0760–0.0760)	1.0

OHIP	Gr. 1 (*n* = 24)	59.4 ± 7.7	21.7 ± 6.2	37.6 ± 9.95	
	Gr. 2 (*n* = 18)	60.4 ± 8.6	25.3 ± 6.8	35.1 ± 10.4	
Difference				2.500 ± 3.164 (−8.8951–3.8951)	0.4341

BI	Gr. 1 (*n* = 24)	—	0.06 ± 0.10	0.07 ± 0.34 (0.0013–0.1387)	0.0459
	Gr. 2 (*n* = 18)		0.13 ± 0.12		

PI	Gr. 1 (*n* = 24)		0.11 ± 0.12	0.09 ± 0.39 (0.0116–0.1684)	0.0255
	Gr. 2 (*n* = 18)		0.20 ± 0.13		

## Data Availability

Data are available on request to the corresponding author.
